# Subcutaneous Single Injection Digital Block with Epinephrine

**DOI:** 10.1155/2012/487650

**Published:** 2011-07-12

**Authors:** Motoki Sonohata, Satomi Nagamine, Kazumasa Maeda, Kenji Ogawa, Hideki Ishii, Kenji Tsunoda, Akihiko Asami, Masaaki Mawatari

**Affiliations:** ^1^Department of Orthopaedic Surgery, Faculty of Medicine, Saga University, 5-1-1 Nabeshima, Saga-shi, Saga 849-8501, Japan; ^2^Department of Orthopaedic Surgery, Saga Insurance Hospital, 3-8-1 Hyogo Minami, Saga-shi, Saga 849-8522, Japan

## Abstract

The aim of this study was to investigate the anesthetic effect and risk of epinephrine for subcutaneous single injection digital block. Either 3.0 mL 1.0% Lidocaine or a 3.0 mL 1.0% Lidocaine with (1 : 100,000) epinephrine was injected into the subcutaneous space at the middle point of the palmar digital crease of the 18 middle fingers of 9 healthy volunteers. The SpO_2_ of the fingers decreased to a maximum of 97. No subjects showed any symptoms of ischemic injury. The time to anesthesia for the fingers was significantly shorter (*P* < 0.05), and the duration of anesthesia was significantly longer (*P* < 0.01) for the fingers in the epinephrine group. In conclusion, a subcutaneous single injection digital blocks with 3.0 mL of 1.0% Lidocaine and (1 : 100,000) epinephrine were safe, reducing the time to the onset of anesthesia, while also markedly prolonging the anesthesia.

## 1. Introduction

Many specialists feel that local anesthesia with epinephrine should not be used for a digital block. Epinephrine is a strong alpha- and beta-receptor agonist and, therefore, results in the activation of alpha-receptors in digital arteries leading to vasoconstriction. The digital arteries are terminal or end arterioles, and this vasoconstriction can lead to ischemia and gangrene [1]. However, a careful review of the literature from 1880 to 2000 revealed that there were only 48 case reports of digital gangrene and necrosis following local anesthesia in the digits, and most of those were published before 1950 [2]. 

In addition, those cases of digital gangrene were associated with procaine and cocaine injection with or without epinephrine. Necrosis has never been reported in patients treated with a commercial lidocaine-epinephrine mixture. Early reports in the second half of the 20th century support the safety of lidocaine with epinephrine in digital anesthesia. Three studies reported no complications after performing digital blocks using local anesthetics with epinephrine in 93 and 98 patients, respectively [3, 4]. 

However, the digital block techniques in those reports were classical digital blocks, using the so-called Oberst procedure. This technique requires at least two injections. Various protocols for single injection digital block have been reported since 1990 [5–8]. In particular, a subcutaneous single injection digital block is simple procedure [8].

The purpose of this study was to investigate the anesthetic effect and risk of epinephrine for subcutaneous single injection digital block.

## 2. Materials and Methods

This study was enrolled on 9 normal, healthy volunteers, who were junior medical residents and whose hands had suffered no nerve trauma or disease. The mean age of the 7 male and 2 female volunteers was 26 (range 20–37) years. The protocol of this study and informed consent conformed to the ethical guidelines of the 1975 Declaration of Helsinki. The study was explained to the volunteers, who signed a consent form and were reimbursed for their time. 

**Figure 1:**
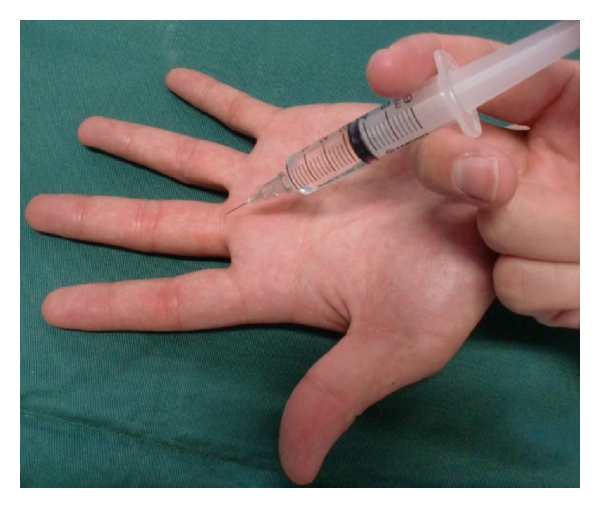
Subcutaneous single injection at the middle point of the palmar digital crease.

A 3.0 mL solution of 1.0% Xylocaine (Lidocaine, AstraZeneca, Japan) and a 3.0 mL solution of 1.0% Xylocaine with (1 : 100,000) epinephrine (Lidocaine, AstraZeneca, Japan) were prepared at room temperature. The solutions were injected into the subcutaneous space at the middle point of the palmar digital crease of the 18 middle fingers of the 9 volunteers using a 5 mL syringe and a 27-gauge needle (Figure 1). A 3.0 mL 1.0% Lidocaine was injected in 9 right middle fingers, and a 3.0 mL 1.0% Lidocaine with (1 : 100,000) epinephrine was injected into the left middle fingers.

The subjects themselves determined the loss of pinprick sensation and its reappearance at their fingertip (palmar distal) every ten seconds up to 60 minutes and each 10 minutes after 60 minutes using the contralateral hand of the injected side. The time to the loss and reappearance of the sensation was measured by the authors using a stopwatch.

The extent of anesthesia was also determined using the pinprick test by the subjects themselves, and they finished at the time when normal sensation was recovered. The extent of anesthesia was recorded by the authors. Each middle finger was divided into 6 zones; the palmar and dorsal areas of the distal segment, middle segment, and proximal segment corresponding to the two surfaces and the three phalangeal segments of the finger [8].

The circulation in the fingers was measured using Pulse Oximeter NPB-40 (COVIDIEN Japan Co., Ltd., Japan) before the digital blocks and at 0.5, 1, 3, 5, 10, 20, 30, 60 minutes after digital blocks. 

The data are presented as the mean ± SD. Student's *t*-test was used to compare the mean variables using the Stat View 5.0 for Windows software package (SAS Institute, USA). The level of significance was set at  *P* < 0.05.

## 3. Results

**Figure 2:**
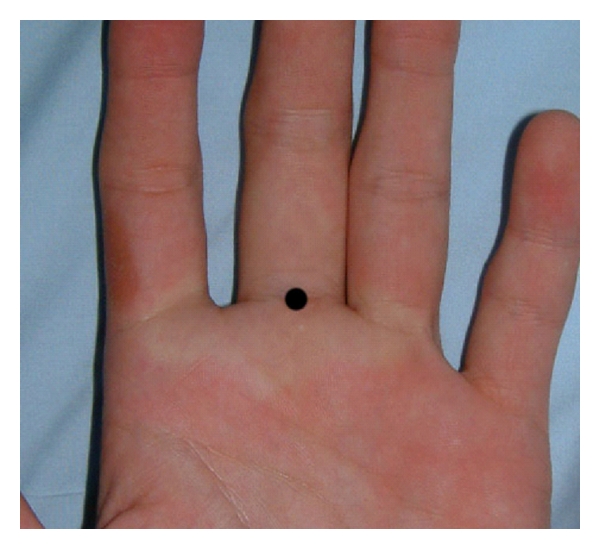
The area around the injection point is completely white. The filled black circle is the injection point.

There was completely white area around the injection site immediately following the injection of 3.0 mL 1.0% Lidocaine with (1 : 100,000) epinephrine into the subcutaneous space at the middle point of the palmar digital crease of the middle fingers (Figure 2). 

**Figure 3:**
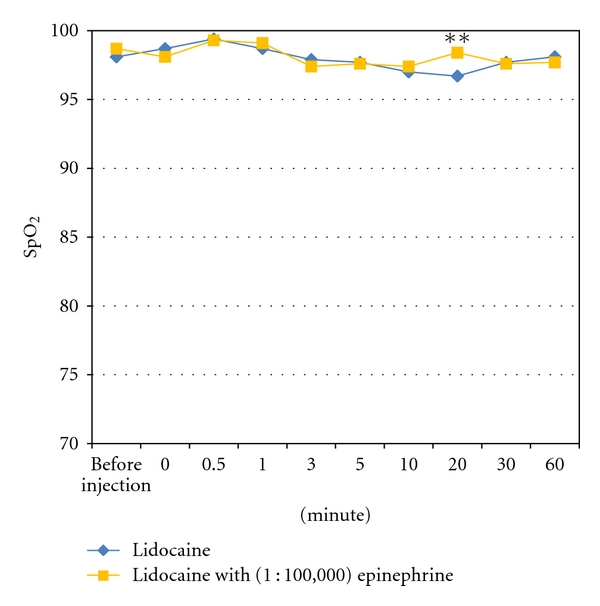
Transitional change of SpO_2_ after a subcutaneous single injection at the middle point of the palmar digital crease. **P < 0.01.

There was no significant difference in the value of SpO_2_ before each digital block (*P* = 0.27). The mean value of SpO_2_ was 96.7 ± 0.98 in the Lidocaine group 20 minutes after the digital block and 98.4 ± 0.95 in the Lidocaine with (1 : 100,000) epinephrine group. There was a significant difference between the two groups (*P* < 0.01). There was no significant difference in the value of SpO_2_ between the groups at any other time points after the digital block (Figure 3).

**Figure 4:**
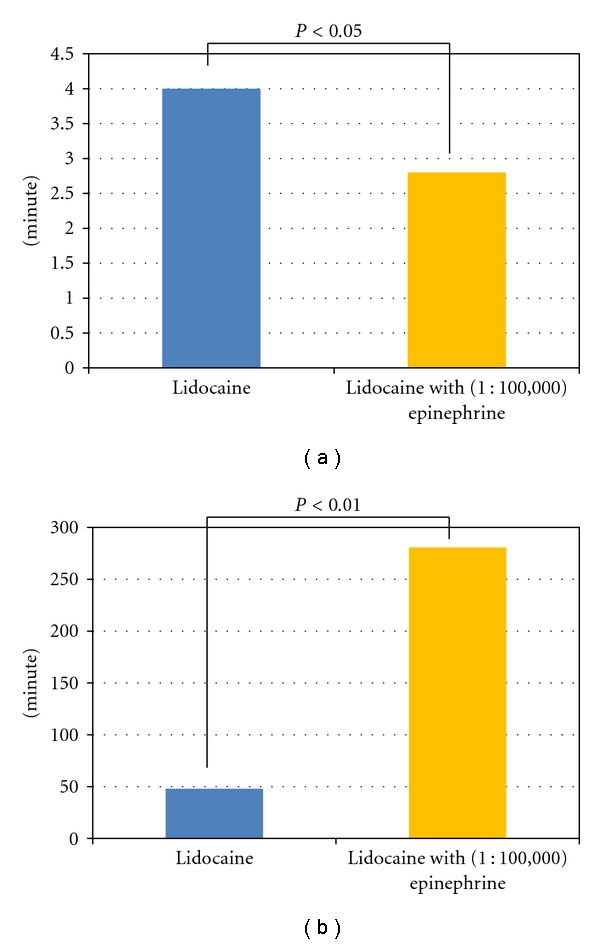
(a) Time to anesthesia. (b) Duration of anesthesia.

The mean time to anesthesia for the fingers in the 3.0 mL 1.0% Lidocaine injection group was 4.0 ± 0.85 minutes, and 2.8 ± 0.83 minutes in the 3.0 mL 1.0% Lidocaine with (1 : 100,000) epinephrine group. There was a significant difference in the time to onset between the two groups (*P* < 0.05). The mean duration of anesthesia in the 1.0% Lidocaine injection group was 48.1 ± 23.5 minutes, and that in the 3.0 mL 1.0% Lidocaine with (1 : 100,000) epinephrine group was 280.7 ± 23.5 minute. There was a significant difference in the duration of anesthesia (*P* < 0.01; Figure 4).

All palmar and dorsal distal and middle segments were anesthetized in both groups. The anesthesia of the dorsal proximal segment was insufficient in all fingers. There were no late complications.

## 4. Discussions

This is the first report to demonstrate that a subcutaneous single injection using Lidocaine with (1 : 100,000) epinephrine was safe for healthy subjects. 

 The skin color around the injection point turned white after a subcutaneous single injection digital block using Lidocaine with (1 : 100,000) epinephrine, due to Epinephrine's marked vasoconstriction, as in previous reports [9]. Sylaidis and Logan [10] reported that the digital artery blood flow rapidly decreases in the first 5 to 10 minutes after a digital block using 2% Lidocaine with (1 : 80,000) epinephrine, and the blood flow returns to normal within 1 hour.

However, the value of SpO_2_ after a subcutaneous single injection digital block using 1% Lidocaine with (1 : 100,000) epinephrine was stable for 60 minutes in the current study. The value of SpO_2_ in the Lidocaine group was only significantly different than the Lidocaine with (1 : 100,000) epinephrine group 20 minutes after digital block; however, the reason for this difference is unclear.

The mean time to anesthesia for the fingers in the Lidocaine with (1 : 100,000) epinephrine group was faster than that in the Lidocaine group. This is an intriguing result. Many reports have noted that epinephrine prolongs anesthesia, and that is consistent with the current findings [10]. However, no report has previously indicated the ability of epinephrine to accelerate anesthesia onset. This accelerated activity could be due to the vasoconstrictive effect of epinephrine, which may have decreased the clearance of the anesthetic and enhanced the efficacy of Lidocaine [11].

The current study demonstrated that a subcutaneous single injection digital block using epinephrine was a safe procedure. However, there may be a possible risk of necrosis with a higher concentration of epinephrine or a greater volume of solution. Digits are very resistant to ischemia [2]. Fitzcharles-Bowe reported that there were no instances of necrosis or skin loss in 59 fingers injected with high-dose (1 : 1,000) epinephrine [9].

 However, all subjects were young healthy volunteers in the current study, and the possible risk of ischemic injury by using epinephrine in patients with preexisting vascular insufficiency cannot be denied.

 In conclusion, the subcutaneous single injection digital blocks of 3.0 mL 1.0% Lidocaine with (1 : 100,000) epinephrine were safe and provided a shorter time to onset of anesthesia and markedly prolonged anesthesia.
